# Efficacy and safety of probiotics as a complementary treatment for urticaria: a systematic review and meta-analysis

**DOI:** 10.3389/fmicb.2025.1634990

**Published:** 2025-12-10

**Authors:** Danni Tan, Ke Liao, Yunfeng Yu, Yuman Yin, Lina Qin, Qin Xiang, Rong Yu

**Affiliations:** 1 School of Traditional Chinese Medicine, Hunan University of Chinese Medicine, Changsha, China; 2 Department of Pediatrics, People’s Hospital of Ningxiang City, Changsha, China; 3 Department of Endocrinology, The First Hospital of Hunan University of Chinese Medicine, Changsha, China

**Keywords:** gut microbiota, meta-analysis, probiotics, trial sequential analysis, urticaria

## Abstract

**Objective:**

The potential of probiotics in the treatment of urticaria has garnered significant attention. However, a systematic assessment of their benefits and risks is lacking. This meta-analysis and trial sequential analysis (TSA) aim to evaluate the efficacy and safety of probiotics as a complementary treatment for urticaria.

**Methods:**

Relevant studies published before April 30, 2025, were retrieved and screened from eight public databases. The basic characteristics, outcome data, and risk of bias of the included studies were recorded. Meta-analysis and TSA were conducted using Review Manager 5.3 and TSA 0.9.5.10 Beta, respectively. Funnel plots and Egger’s test were employed to assess publication bias, while the Grading of Recommendations, Assessment, Development, and Evaluation (GRADE) system was used to evaluate the certainty of evidence.

**Results:**

A total of 11 clinical trials involving 1,014 participants were included in this study. The meta-analysis revealed that, compared to antihistamines alone, the combination of probiotics and antihistamines significantly improved the urticaria relief rate (URR) (odds ratio [OR] 2.90, 95% confidence interval [CI] 1.97 to 4.28, *p* < 0.00001) and interferon-gamma (IFN-γ) levels (mean difference [MD] 8.12, 95% CI 6.95 to 9.30, *p* < 0.00001). Additionally, it reduced the urticaria activity score over 7 days (UAS7) (MD −3.29, 95% CI −3.28 to −2.75, *p* < 0.00001), the dermatology life quality index (DLQI) (MD −2.95, 95% CI −3.21 to −2.69, *p* < 0.00001), interleukin-10 (IL-10) levels (MD −1.47, 95% CI −2.01 to −0.93, *p* < 0.00001), adverse event rate (AER) (OR 0.39, 95% CI 0.20 to 0.77, *p* = 0.007), and recurrence rate (OR 0.29, 95% CI 0.14 to 0.60, *p* = 0.0008). The TSA confirmed the conclusiveness of the aforementioned meta-analysis results. Egger’s test indicated no significant publication bias for these outcomes.

**Conclusion:**

Probiotics not only improve clinical symptoms in patients with urticaria but may also reduce the incidence of adverse events and recurrence rates. This highlights the potential of probiotics as a complementary treatment for urticaria. However, due to the risk of bias in the existing studies, these findings need to be validated through high-quality clinical trials.

**Systematic review registration:**

https://www.crd.york.ac.uk/PROSPERO/view/CRD420251232590, CRD420251232590.

## Graphical abstract

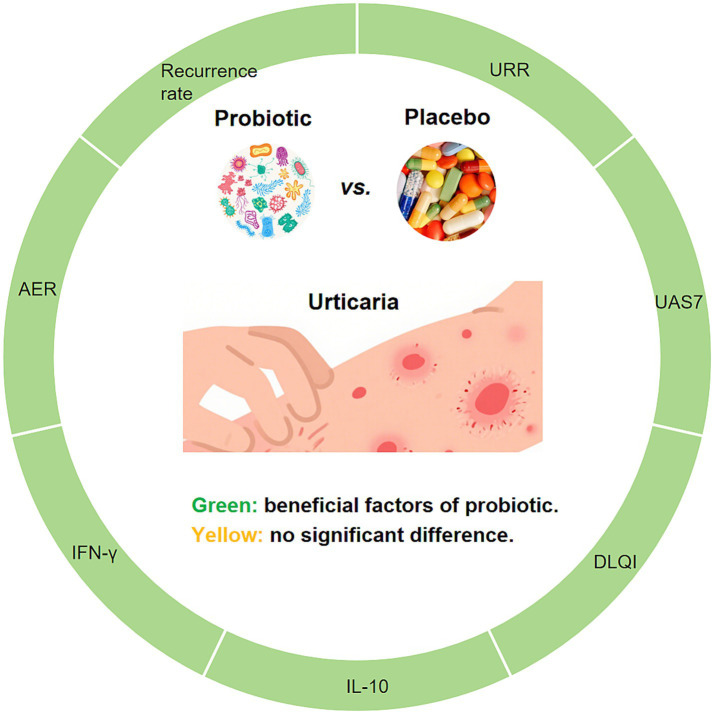

## 1 Introduction

Urticaria is a prevalent mast cell-mediated inflammatory skin disease characterized by wheals and/or angioedema (Church et al., 2018). Epidemiological surveys indicate that the point prevalence of chronic urticaria is 0.7%, with higher rates in Latin America and Asia than in other regions (Fricke et al., 2020). A nationwide cross-sectional study estimated the lifetime prevalence of chronic urticaria in China to be 7.30%, with a prevalence of 8.26% in females and 6.34% in males (Li et al., 2022). Recurrent wheals accompanied by pruritus or erythema are the primary symptoms of urticaria, which seriously affect the patients’ quality of life and increase the risk of depression, anxiety, and sleep disorders (Tawil et al., 2023; Sánchez-Borges et al., 2021). Urticaria can be classified as acute or chronic depending on symptom duration, with chronic urticaria persisting for more than 6 weeks. Chronic urticaria often presents with fluctuating disease activity, with symptoms lasting for months or even years, and a relapsing–remitting course that imposes a substantial psychological and quality-of-life burden on affected individuals (Radonjic-Hoesli et al., 2018). The pathophysiology of urticaria involves complex immune mechanisms, where activated mast cells and basophils degranulate in response to immunologic or non-immunologic triggers, releasing histamine, leukotrienes, and other proinflammatory mediators that cause vasodilation and increased vascular permeability (Hennino et al., 2006). Additionally, dysregulation of both innate and adaptive immune responses, including increased levels of Th2 cytokines, autoimmunity, and altered gut-skin axis interactions, have been implicated in the progression of urticaria (Hennino et al., 2006; Cai et al., 2024). Before the approval of Food and Drug Administration (FDA) on omalizumab in 2014 (Maurer et al., 2013), H1 receptor antagonists were the only drugs sanctioned for the treatment of chronic urticaria (Maurer et al., 2011). However, 60% of patients with chronic urticaria fail to achieve effective symptom control at the standard licensed dose of H1 receptor antagonists (Guillén-Aguinaga et al., 2016; Staevska et al., 2010). Even after receiving treatment with 100 mg or 150 mg of omalizumab for over six months, approximately 30% of patients continue to experience significant symptoms (Metz et al., 2020). Considering the limitations of existing treatments, actively exploring and developing other complementary treatments has become the crucial for overcoming the challenges associated with urticaria.

With the progress of microbiology research, there is a growing recognition that gut microbiota disturbance is a potential pathogenesis and aggravating factors of urticaria (Luo et al., 2022; Wang et al., 2020; Song et al., 2022). Studies supporting this view have pointed out that patients with urticaria exhibit a distinct gut microbiota characterized by a reduction in beneficial intestinal bacteria and an increase in opportunistic pathogens compared to healthy individuals (Liu et al., 2021). Another study also reported similar findings, highlighting that the intestinal microbiome of patients with chronic spontaneous urticaria is marked by reduced diversity, reduced relative abundance of butyrate-producing intestinal bacteria, and elevated levels of conditional pathogens (Zhu et al., 2024). Further studies have indicated that in patients with chronic spontaneous urticaria, an increased abundance of *Bacteroidetes* and a decreased count of *Bacillota* are associated with higher disease activity and poorer disease control (Podder et al., 2025). This microbial imbalance may exacerbate eosinophil-driven skin inflammation (Zhu et al., 2024) through impaired intestinal barrier function and abnormal Th2/Th17 immune responses (Chen et al., 2018). Therefore, gut microbiota disturbance is a potential mechanism triggering urticaria, suggesting that probiotic supplementation may improve the prognosis of urticaria. Li et al. (2013) conducted the first clinical trial comparing the effects of loratadine combined with probiotics versus loratadine alone in the treatment of chronic urticaria. The results showed that loratadine combined with a multi strain probiotic preparation, comprising *Bifidobacterium infantis*, *Lactobacillus acidophilus*, *Enterococcus faecalis,* and *Bacillus cereus*, significantly reduced pruritus and wheal formation, and increased serum levels of interferon-gamma (IFN-γ), compared to loratadine without probiotics. Subsequently, a recent study has shown that, when compared with ebastine, a second generation H1-antihistamine, the combination of *Lactobacillus reuteri* and ebastine can significantly alleviate clinical symptoms and enhance the quality of life scores among patients suffering from chronic urticaria (Can et al., 2024). Notably, a meta-analysis by Fu et al. (2023) suggested that probiotics combined with antihistamines may enhance the therapeutic efficacy in urticaria treatment. However, this meta-analysis included studies on other allergic skin conditions such as eczema and atopic dermatitis, which introduced considerable clinical heterogeneity. Moreover, it lacked a clear definition of “therapeutic effect” and did not report internationally recognized outcome measures such as Urticaria Activity Score over 7 days (UAS7), raising concerns about the reliability and generalizability of its findings. Therefore, a more rigorous and targeted meta-analysis focusing specifically on urticaria is urgently needed to comprehensively assess the efficacy and safety of probiotic interventions.

In this study, we conducted a thorough search of the relevant literature in this domain and used meta-analysis and trial sequential analysis (TSA) to evaluate the efficacy and safety of probiotics as a complementary treatment for urticaria. By systematically investigating the potential benefits and risks of probiotics, this study hopes to provide evidence-based evidence for the microbial-assisted treatment and management of urticaria (see Graphical abstract).

## 2 Methods

This meta-analysis was conducted in accordance with the Preferred Reporting Items for Systematic Reviews and Meta-Analyses (PRISMA) guidelines (Page et al., 2021). The aim was to evaluate the efficacy and safety of probiotics as a complementary treatment for urticaria.

### 2.1 Inclusion and exclusion criteria

Inclusion criteria: (i) Participants: Patients diagnosed with urticaria, without gender, age, race, or disease duration restrictions. (ii) Interventions: The experimental group received probiotics in combination with standard anti-allergy treatment. (iii) Comparisons: The control group only received standard anti-allergy treatment (mainly antihistamines). (iv) Outcomes: The primary outcomes were the urticaria relief rate (URR) and the UAS7. Secondary outcomes included the dermatology life quality index (DLQI), interleukin-10 (IL-10) levels, IFN-γ levels, the recurrence rate, and the adverse event rate (AER). (v) Study design: Only randomized controlled trials (RCTs) and case–control studies were included in this review.

Exclusion criteria: (i) Duplicate publications: Studies reporting the same data in multiple publications were excluded to avoid redundancy. (ii) Abstract format publications: Studies that were published solely as abstracts or conference proceedings without full-text availability were excluded. (iii) Incomplete or unclear data: Studies that lacked sufficient data or presented unclear or ambiguous outcome effects, making it impossible to assess their relevance or quality, were excluded. (iv) Inappropriate statistical methods: Studies that employed incorrect statistical methods, and for which corrections could not be made, were excluded from the analysis.

### 2.2 Literature search strategy

A comprehensive literature search was conducted across seven databases: PubMed, Embase, Cochrane Library, Web of Science, China National Knowledge Infrastructure (CNKI^1^), Wanfang Data,^2^ and the China Science and Technology Journal Database (VIP^3^). In addition, ClinicalTrials.gov was searched to identify unpublished or ongoing trials and minimize publication bias. CNKI, Wanfang, and VIP are widely used Chinese academic databases. While these databases are region-specific and may include gray literature, they provide unique and valuable data not indexed in international databases. To ensure data quality and minimize bias, all studies retrieved from these Chinese sources were independently screened, translated, and assessed for methodological quality by two reviewers. These quality control measures help ensure that the inclusion of Chinese-language studies does not compromise the robustness or generalizability of the meta-analysis and supports the reproducibility of the search strategy for researchers worldwide.

The search fields were set to Title/Abstract or Topic, and the following search strategy was applied: (Urticarias OR Urticaria OR Urticarial Wheal OR Urticarial Wheals) AND (Probiotic OR Probiotics OR *Lactobacillus acidophilus* OR *Lactobacillus amylovorus* OR *Lactobacillus* OR *Bacillus bifida* OR *Bacillus* OR *Clostridium butyricum* OR *Streptococcus thermophiles* OR *Enterococcus* OR *Bifidobacterium* OR *Bifidobacteria* OR *Yeast* OR *Saccharomyces cerevisiae* OR *Saccharomyces italicus* OR *Saccharomyces oviformis* OR *S cerevisiae* OR *S. cerevisiae* OR *Saccharomyces u*var*um* var. *melibiosus* OR *Candida robusta* OR *Saccharomyces capensis* OR *Natto Bacteria* OR *Lactic acid bacteria*). To maintain search specificity and avoid retrieving studies unrelated to urticaria, we intentionally excluded broader or ambiguous terms such as “hives,” “dermatological hypersensitivity,” “next-generation probiotics,” or “gut microbiota modulation.” However, relevant concepts falling under these broader categories were captured through manual screening of reference lists and related systematic reviews to ensure comprehensiveness.

The above search covered literature published from database inception through April 30, 2025, with no language restrictions. Non-English articles, including all Chinese studies, were translated into English as necessary. All translations were independently reviewed and cross-checked by two bilingual researchers with medical backgrounds to ensure consistency and technical accuracy. Any ambiguities or potential mistranslations were resolved through consensus discussion and direct comparison with the original Chinese texts. The complete search strategies for each database are provided in Supplementary Table S1.

### 2.3 Study selection process

All retrieved records were imported into EndNote version X9 (Clarivate Analytics). Duplicate entries were removed automatically and verified manually. Two independent reviewers (DT and YY), both of whom received formal training in evidence-based medicine and systematic review methodology, screened the titles and abstracts according to the predefined inclusion and exclusion criteria. Full-text articles of potentially eligible studies were then retrieved and assessed independently by the same reviewers. Any disagreements during the selection process were resolved through discussion or by consulting a third reviewer (QX), an expert in clinical research and systematic reviews. The study selection process was documented using a PRISMA flow diagram.

### 2.4 Data extraction

A standardized data extraction form was developed (Yu et al., 2023; Deng et al., 2024). Data collected included: basic information (authors, publication year, country), study characteristics (design, sample size), participant demographics (age, gender, course of disease), details of the intervention and control groups (intervention treatment used, conventional treatment used, type of probiotics), outcomes assessed, funding sources, and potential conflicts of interest. Two reviewers extracted data independently; any discrepancies were discussed and resolved. When necessary, authors were contacted for missing or unclear data.

### 2.5 Risk of bias assessment

The methodological quality of included studies was evaluated independently by two reviewers using the Cochrane Risk of Bias tool. Domains assessed included sequence generation, allocation concealment, blinding of participants and personnel, blinding of outcome assessment, incomplete outcome data, selective reporting, and other biases. Each domain was rated as “low,” “high,” or “unclear” risk. An overall risk of bias judgment was provided. Any disagreements were resolved by discussion or involving a third reviewer.

### 2.6 Data synthesis and statistical analysis

Review Manager version 5.3 (Cochrane Collaboration^4^) (Higgins et al., 2019), a software specifically designed for conducting systematic reviews and meta-analyses, was used to perform our analyses. For dichotomous outcomes, such as the URR, recurrence rate, and AER, odds ratios (ORs) with 95% confidence intervals (CIs) were calculated. For continuous outcomes, including the UAS7, DLQI, IL-10, and IFN-γ, mean differences (MDs) or standardized mean differences (SMDs) with 95% CIs were used, depending on measurement tools (Yu et al., 2024). Heterogeneity across studies was assessed and quantified with the I^2^ statistic. An *I*^2^ < 50% indicated low to moderate heterogeneity and justified using a fixed-effects model; otherwise, a random-effects model was applied. When heterogeneity remained high or data were incompatible for pooling, a narrative synthesis was provided (Yu et al., 2022).

Sensitivity analyses were performed to assess the robustness of the results. The influence of studies with high risk of bias, small sample sizes, or extreme effect sizes was tested by excluding each study one at a time. If the results remained consistent across the various sensitivity analyses, this indicates that the overall findings are robust and reliable. Furthermore, subgroup analyses based on clinical factors such as country, female ratio, average age, disease duration, probiotic quantity, and treatment duration were pre-assigned to assess the impact of clinical heterogeneity on the primary outcomes.

To control for random errors and determine the reliability of the cumulative evidence, TSA was performed using the TSA 0.9.5.10 Beta software. The information size was calculated based on an anticipated effect size, an alpha level of 5%, and a power of 80%. TSA sequential monitoring boundaries were applied to assess whether the cumulative evidence was sufficient to draw firm conclusions or if additional studies were necessary. If the cumulative Z-curve crossed the monitoring boundary, the result was considered conclusive. This approach helps to prevent premature or spurious findings by adjusting for the risk of type I and type II errors in the meta-analysis.

### 2.7 Publication bias

Publication bias was assessed visually using funnel plots and quantitatively through Egger’s test. Asymmetry in the funnel plot suggested the potential for publication bias, and a *p*-value of < 0.05 from Egger’s test was considered significant.

### 2.8 Certainty of evidence

The certainty of the evidence for each outcome was assessed using the Grading of Recommendations, Assessment, Development, and Evaluation (GRADE) system. This evaluation considered factors such as risk of bias, inconsistency, indirectness, imprecision, and publication bias. Each outcome was graded as high, moderate, low, or very low quality, which informed the confidence in the pooled estimates and the recommendations derived from them (Zhang et al., 2019).

## 3 Results

### 3.1 Study selection

**Figure 1:**
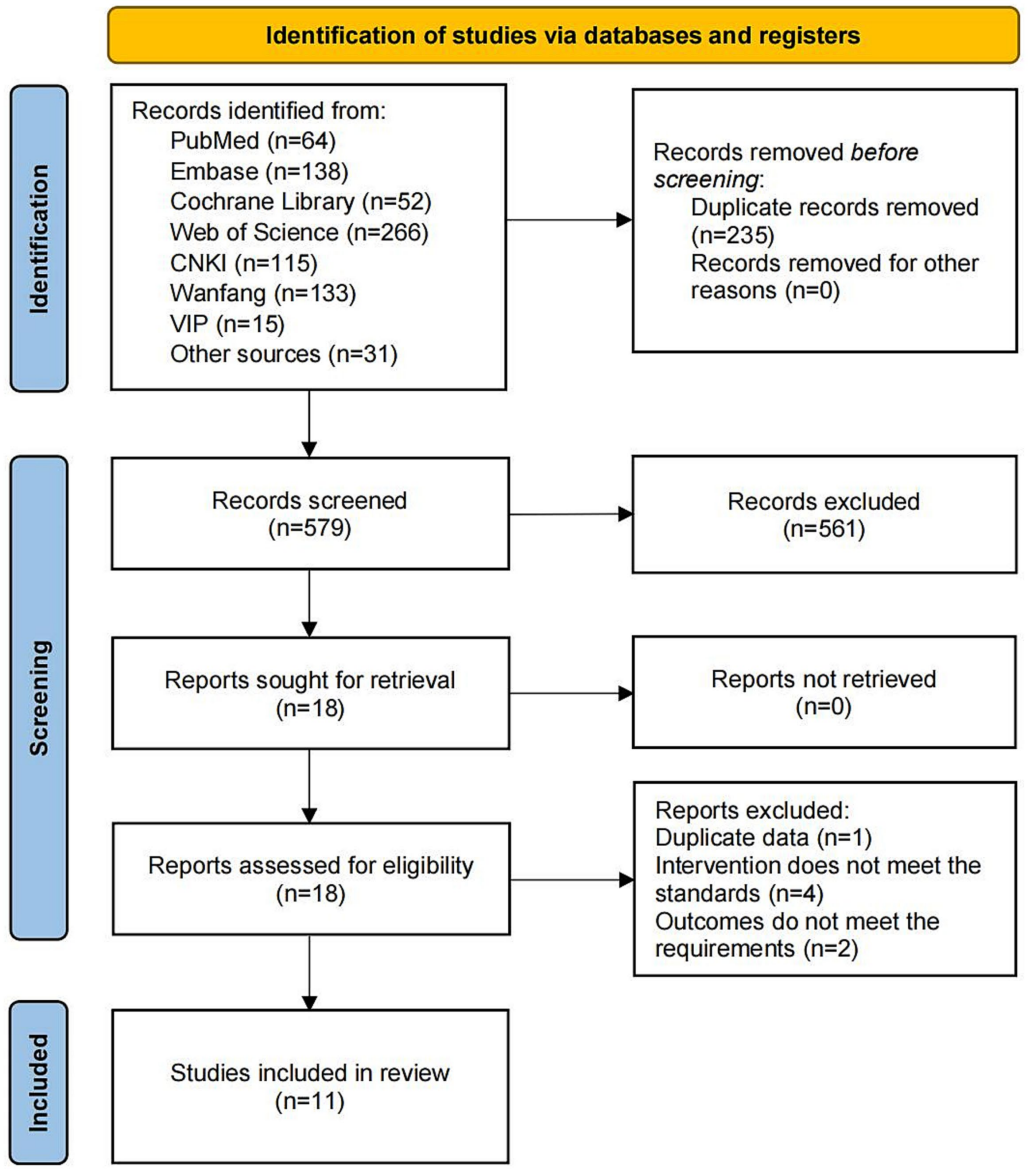
PRISMA process diagram.

The literature search was conducted across multiple databases, yielding a total of 64 records from PubMed, 138 from Embase, 52 from the Cochrane Library, 266 from Web of Science, 115 from CNKI, 133 from Wanfang, 15 from VIP, and 31 from other sources. In total, 799 records were identified. After removing duplicate records (*n* = 235), the remaining records were screened based on titles and abstracts, resulting in the exclusion of 561 records. Subsequently, full-text articles were assessed for eligibility, leading to the exclusion of seven records that did not meet the predefined criteria: one record was excluded due to duplicate data, four records did not meet the intervention standards, and two records did not fulfill the outcome requirements. Ultimately, 11 studies (Li et al., 2013; Can et al., 2024; Li, 2022; Dabaghzadeh et al., 2023; Bi et al., 2021; Atefi et al., 2022; Ye et al., 2018; Fu et al., 2020; Wang et al., 2023; Liang, 2021; Chen et al., 2013) were included in this meta-analysis. The flowchart is shown in Figure 1.

### 3.2 Basic characteristics of included studies

A total of 11 clinical trials (Li et al., 2013; Can et al., 2024; Li, 2022; Dabaghzadeh et al., 2023; Bi et al., 2021; Atefi et al., 2022; Ye et al., 2018; Fu et al., 2020; Wang et al., 2023; Liang, 2021; Chen et al., 2013) and 1,014 participants were included in this meta-analysis, consisting of 10 RCTs (Li et al., 2013; Li, 2022; Dabaghzadeh et al., 2023; Bi et al., 2021; Atefi et al., 2022; Ye et al., 2018; Fu et al., 2020; Wang et al., 2023; Liang, 2021; Chen et al., 2013) and 1 case–control study (Can et al., 2024). The publication years of the included studies spanned from 2013 to 2024, with those published in the past 5 years accounting for 72.7%. Eight studies were from China, two were from Iran, and one was from Turkey. The proportion of females in the included studies ranged from 37.5 to 81.6%, and the average age ranged from 5.9 years to 39.5 years. Three studies used probiotic preparations with a single strain, while 8 studies used probiotic preparations with multiple strains. The shortest treatment course was 2 weeks, and the longest was 8 weeks. The basic characteristics are shown in Table 1.

**Table 1:** Basic characteristics of included studies.

Study	Country	Sample (E/C)	Female (%)	Age (years)	Disease duration (months)	Intervention	Comparison	Strain	Treatment duration (weeks)
Atefi et al. (2022)	Iran	21/21	71.4	38.4	4.8	LactoCare® and any two of cetirizine 20 mg/d, desloratadine 10 mg/d, and fexofenadine 360 mg/d	Any two of cetirizine 20 mg/d, desloratadine 10 mg/d, and fexofenadine 360 mg/d	*Lactobacillus rhamnosus*, *Lactobacillus casei*, *Lactobacillus acidophilus*, *Bifidobacterium breve*, *Lactobacillus bulgaricus*, *Bifidobacterium longum*, *Streptococcus thermophilus*	8
Bi et al. (2021)	China	104/102	54.9	8.6	1.5–5.8	Yimingjia® 3 g/d and desloratadine dry suspension 2.5 g/d	Placebo and desloratadine dry suspension 2.5 g/d	*Lactobacillus gasseri*, *Lactobacillus salivarius*, *Lactobacillus johnsonii*, *Lactobacillus paracasei*, *Lactobacillus reuteri*, *Bifidobacterium animalis*	4
Can et al. (2024)	Türkiye	30/30	80.0	39.5	28.6	*Lactobacillus reuteri* 5 drops/d and ebastine 10 mg/d	Ebastine 10 mg/d	*Lactobacillus reuteri*	4
Chen et al. (2013)	China	34/32	53.0	8.9	1.2–72.0	Siliankang® 3.0 g/d and cetirizine 1 mL/d	Cetirizine 1 ml/d	*Bifidobacterium, Lactobacillus*, *Enterococcus* and *Bacillus cereus*	2
Dabaghzadeh et al. (2023)	Iran	20/18	81.6	25.7	/	Femilact® capsule and cetirizine 20 mg/d	Placebo and cetirizine 20 mg/d	*Lactobacilluses* (*Casei, Acidophilus*, *Bulgaricus*, *Rhamnosus*), *Bifidobacterium* (*Breve* and *Longum*), *Streptococcus Thermophilus*	8
Fu et al. (2020)	China	32/32	37.5	35.9	22.8	Selenious yeast 200 μg/d and rupatadine 10 mg/d	Rupatadine 10 mg/d	*Saccharomyces cerevisiae* Hansen	4
Li et al. (2013)	China	43/43	59.3	9.8	10.2	Siliankang® 2.0 g/d and loratadine 5–10 mg/d	Loratadine 5–10 mg/d	B*ifidobacterium, Lactobacillus*, *Enterococcus* and *Bacillus cereus*	4
Li et al. (2022)	China	30/30	41.7	/	/	Siliankang® 3.0 g/d and cetirizine 1 ml/d	Cetirizine 1 ml/d	*Bifidobacterium*, *Lactobacillus*, *Enterococcus* and *Bacillus cereus*	2
Liang (2021)	China	35/33	39.7	5.9	/	Aiminkang® 4 g/d and loratadine 10 mg/d	Loratadine 10 mg/d	*Lacticaseibacillus paracasei*, *Limosilactobacillus fermentum*, *Limosilactobacillus reuteri*, *Bifidobacterium animalis subsp.lactis*, *Lactiplantibacillus plantarum*, *Lactobacillus*, *Lacticaseibacillus rhamnosus*	4
Wang et al. (2023)	China	120/120	42.5	39.3	12.0	Meichangan® 1.0 g/d, loratadine 10 mg/d, and MYG 10 g/d	Loratadine 10 mg/d and MYG 10 g/d	*Enterococcus faecium and Bacillus subtilis*	4
Ye et al. (2018)	China	42/42	52.4	37.2	24.4	Selenious yeast 200 μg/d and cetirizine 10 mg/d	Cetirizine 10 mg/d	*Saccharomyces cerevisiae* Hansen	8

E, experimental group; C, control group. There were no significant differences in female ratio, average age, or disease duration between the experimental and control groups in each included study.

### 3.3 Risk of bias

The risk of bias assessment was conducted across several domains for the included studies. In terms of random sequence generation, one study (Can et al., 2024) was deemed to be at high risk due to the absence of a random design, while four studies (Li et al., 2013; Li, 2022; Liang, 2021; Chen et al., 2013) were classified as having unclear risk because they mentioned randomization without providing sufficient details. The remaining six studies (Dabaghzadeh et al., 2023; Bi et al., 2021; Atefi et al., 2022; Ye et al., 2018; Fu et al., 2020; Wang et al., 2023) were assessed as low risk, as they described detailed randomization methods.

Regarding allocation concealment, one study (Can et al., 2024) was rated as high risk due to the lack of allocation concealment, while seven studies (Li et al., 2013; Li, 2022; Ye et al., 2018; Fu et al., 2020; Wang et al., 2023; Liang, 2021; Chen et al., 2013) were classified as having unclear risk because they did not describe the allocation concealment process. Three studies (Dabaghzadeh et al., 2023; Bi et al., 2021; Atefi et al., 2022) were assessed as low risk, as they implemented allocation concealment through software.

In the domain of blinding of participants and personnel, two studies (Can et al., 2024; Atefi et al., 2022) were classified as high risk because they were explicitly identified as open-label trials, and seven studies (Li et al., 2013; Li, 2022; Ye et al., 2018; Fu et al., 2020; Wang et al., 2023; Liang, 2021; Chen et al., 2013) were rated as having unclear risk due to insufficient detail regarding blinding procedures. However, two studies (Dabaghzadeh et al., 2023; Bi et al., 2021) were assessed as low risk, as they clearly employed a placebo control.

**Figure 2:**
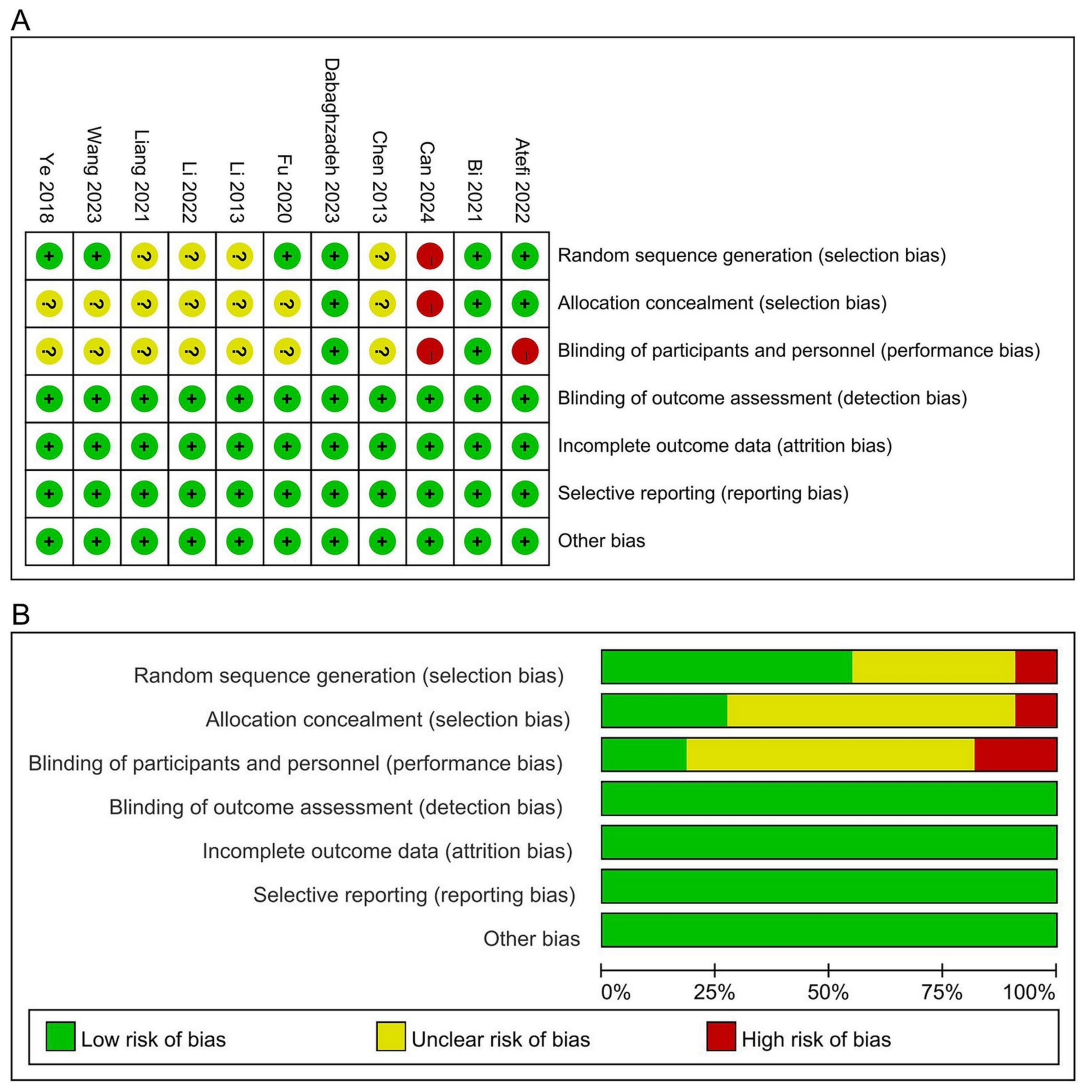
Risk of bias: **(A)** Risk of bias graph; **(B)** Risk of bias summary.

For blinding of outcome assessment, all studies (Li et al., 2013; Can et al., 2024; Li, 2022; Dabaghzadeh et al., 2023; Bi et al., 2021; Atefi et al., 2022; Ye et al., 2018; Fu et al., 2020; Wang et al., 2023; Liang, 2021; Chen et al., 2013) were rated as low risk as they utilized objective outcome measures. For incomplete outcome data, all studies (Li et al., 2013; Can et al., 2024; Li, 2022; Dabaghzadeh et al., 2023; Bi et al., 2021; Atefi et al., 2022; Ye et al., 2018; Fu et al., 2020; Wang et al., 2023; Liang, 2021; Chen et al., 2013) were classified as low risk, as the dropout rates were below 20%. Additionally, all studies (Li et al., 2013; Can et al., 2024; Li, 2022; Dabaghzadeh et al., 2023; Bi et al., 2021; Atefi et al., 2022; Ye et al., 2018; Fu et al., 2020; Wang et al., 2023; Liang, 2021; Chen et al., 2013) reported predefined outcomes, with no evidence of reporting bias or other biases identified. Overall, the overall risk of bias was low in two studies (Dabaghzadeh et al., 2023; Bi et al., 2021), some concerns in seven studies (Li et al., 2013; Li, 2022; Ye et al., 2018; Fu et al., 2020; Wang et al., 2023; Liang, 2021; Chen et al., 2013), and high in two studies (Can et al., 2024; Atefi et al., 2022), as shown in Figure 2.

### 3.4 Meta-analysis and sensitivity analysis

#### 3.4.1 URR

**Figure 3:**
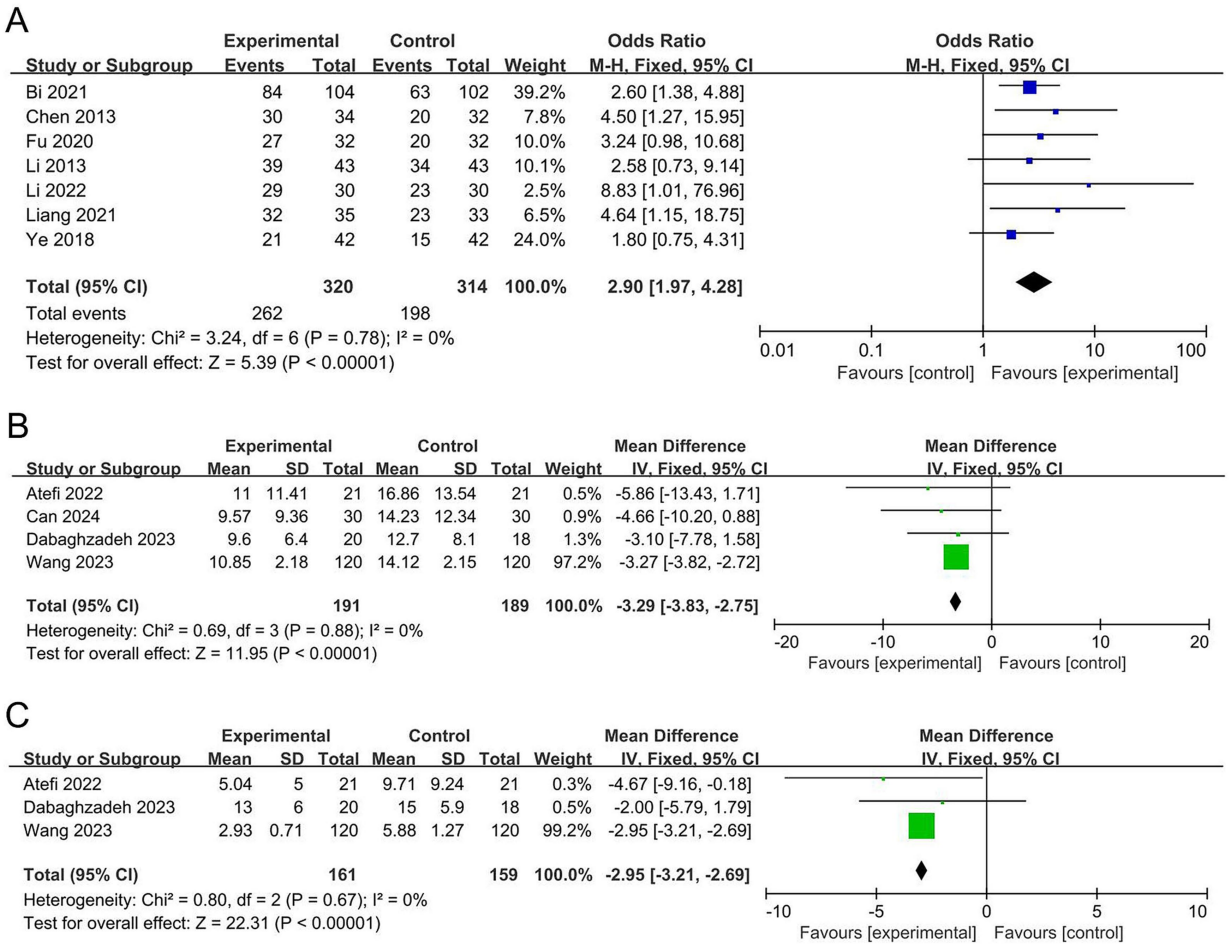
Forest plots of the meta-analysis on URR, UAS7, and DLQI: **(A)** URR; **(B)** UAS7; **(C)** DLQI. URR, urticaria relief rate; UAS7, urticaria activity score over 7 days; DLQI, dermatology life quality index.

The meta-analysis for URR included seven studies (Li et al., 2013; Li, 2022; Bi et al., 2021; Ye et al., 2018; Fu et al., 2020; Liang, 2021; Chen et al., 2013) with a total of 634 participants. The I^2^ statistic indicated no significant heterogeneity (*I*^2^ = 0). The results demonstrated that the probiotic group significantly improved the URR by 190% compared to the control group (OR 2.90, 95% CI 1.97 to 4.28, *p* < 0.00001), as shown in Figure 3A. The sensitivity analysis revealed that the significance of URR did not change after the removal of any individual study, indicating that the results are robust.

#### 3.4.2 UAS7

The meta-analysis for UAS7 included four studies (Can et al., 2024; Dabaghzadeh et al., 2023; Atefi et al., 2022; Wang et al., 2023) with a total of 380 participants. The I^2^ statistic indicated no significant heterogeneity (*I*^2^ = 0). The results revealed that the probiotic group significantly reduced UAS7 by 3.29 points compared to the control group (MD −3.29, 95% CI −3.28 to −2.75, *p* < 0.00001), as shown in Figure 3B. The sensitivity analysis showed that the significance of UAS7 did not change when any individual study was removed, indicating that the results are robust.

#### 3.4.3 DLQI

The meta-analysis for DLQI included three studies (Dabaghzadeh et al., 2023; Atefi et al., 2022; Wang et al., 2023) with a total of 320 participants. The I^2^ statistic indicated no significant heterogeneity (*I*^2^ = 0). The results showed that the probiotic group significantly decreased the DLQI by 2.95 points compared to the control group (MD −2.95, 95% CI −3.21 to −2.69, *p* < 0.00001), as shown in Figure 3C. The sensitivity analysis indicated that the significance of DLQI did not change when any single study was removed, suggesting that the results are robust.

#### 3.4.4 IL-10

**Figure 4:**
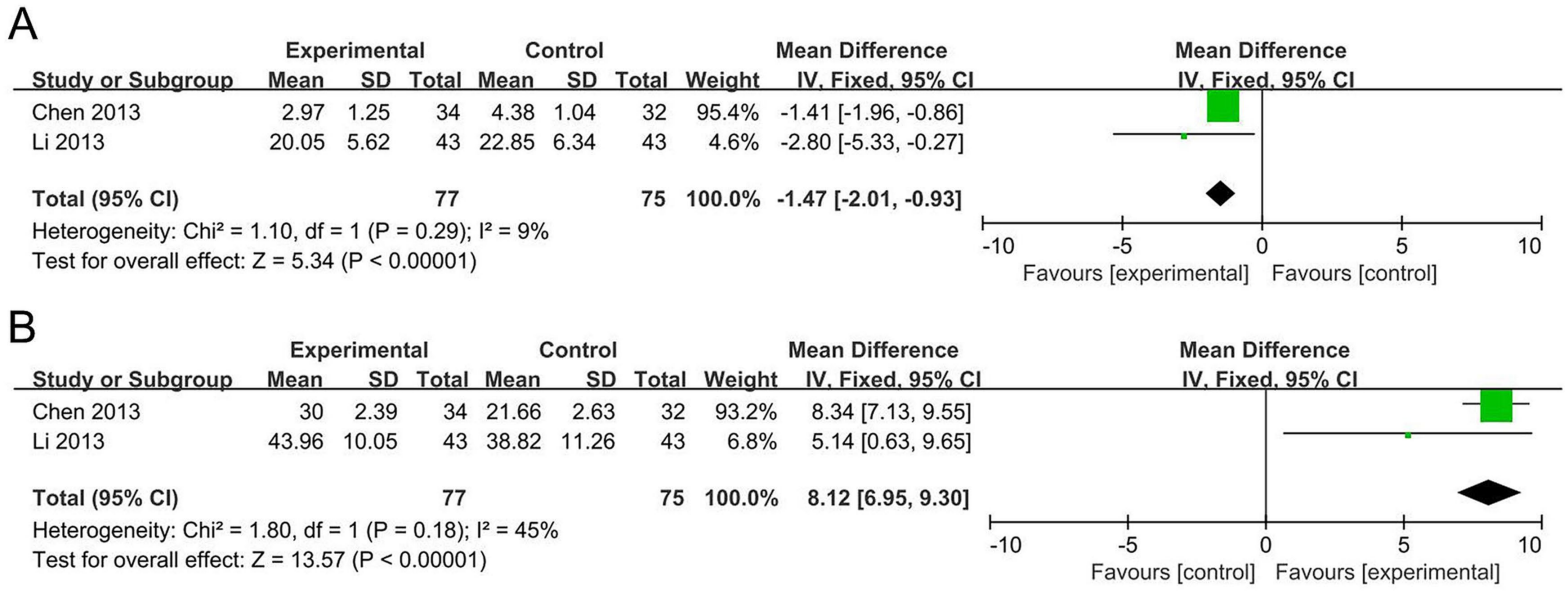
Forest plots of the meta-analysis on serum IL-10 and IFN-γ levels: **(A)** Serum IL-10 levels; **(B)** Serum IFN-γ levels. IL-10, interleukin-10; IFN-γ, interferon-gamma.

The meta-analysis for IL-10 included two studies (Li et al., 2013; Chen et al., 2013) with a total of 152 participants. The I^2^ statistic indicated mild heterogeneity (*I*^2^ = 9%). The results revealed that the probiotic group significantly decreased IL-10 levels by 1.47 pg./mL compared to the control group (MD −1.47, 95% CI −2.01 to −0.93, *p* < 0.00001), as shown in Figure 4A. The sensitivity analysis demonstrated that the significance of IL-10 did not change after removing any single study, indicating that the results are robust.

#### 3.4.5 IFN-γ

The meta-analysis for IFN-γ included two studies (Li et al., 2013; Chen et al., 2013) with a total of 152 participants. The I^2^ statistic indicated moderate heterogeneity (*I*^2^ = 45%). The results showed that the probiotic group significantly increased IFN-γ levels by 8.12 pg./mL compared to the control group (MD 8.12, 95% CI 6.95 to 9.30, *p* < 0.00001), as shown in Figure 4B. The sensitivity analysis revealed that the significance of IFN-γ did not change when any individual study was removed, indicating that the results are robust.

#### 3.4.6 AER

**Figure 5:**
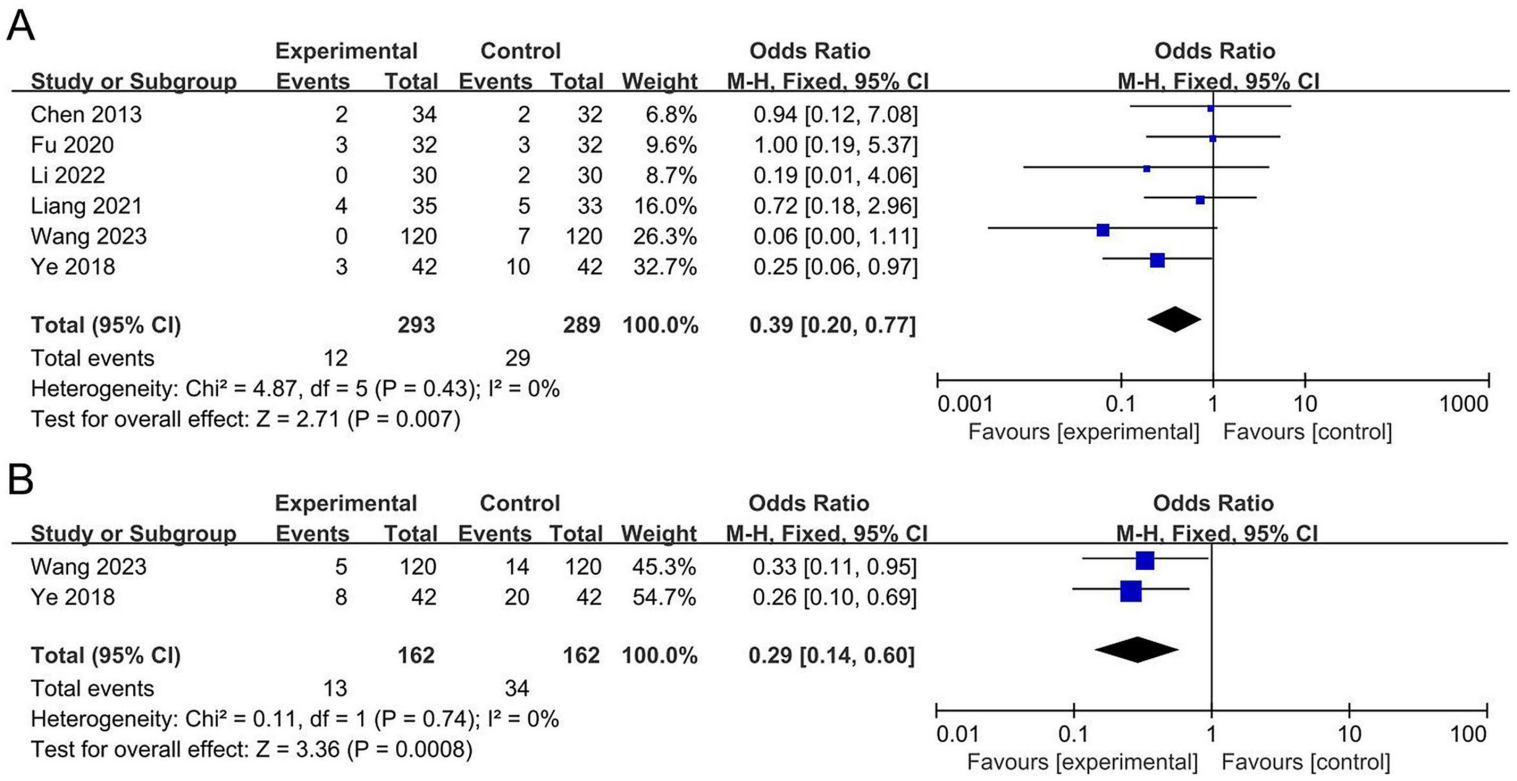
Forest plots of the meta-analysis on the AER and recurrence rate: **(A)** AER; **(B)** Recurrence rate. AER, adverse event rate.

The meta-analysis for AER included six studies (Li, 2022; Ye et al., 2018; Fu et al., 2020; Wang et al., 2023; Liang, 2021; Chen et al., 2013) with a total of 582 participants. The I^2^ statistic indicated no significant heterogeneity (*I*^2^ = 0). The results demonstrated that the probiotic group significantly reduced the AER by 61% compared to the control group (OR 0.39, 95% CI 0.20 to 0.77, *p* = 0.007), as shown in Figure 5A. However, the sensitivity analysis showed that after removing the study by Wang et al. (2023), the difference in AER between the two groups became non-significant (OR 0.50, 95% CI 0.24 to 1.05, *p* = 0.07), suggesting that the meta-analysis results of AER are not robust.

#### 3.4.7 Recurrence rate

The meta-analysis for recurrence rate included two studies (Ye et al., 2018; Wang et al., 2023) with a total of 324 participants. The I^2^ statistic indicated no significant heterogeneity (*I*^2^ = 0). The results revealed that the probiotic group significantly reduced the recurrence rate by 71% compared to the control group (OR 0.29, 95% CI 0.14 to 0.60, *p* = 0.0008), as shown in Figure 5B. The sensitivity analysis showed that the significance of the recurrence rate did not change when any individual study was removed, indicating that the results are robust.

### 3.5 Subgroup analysis

Subgroup analyses were used to examine the effects of clinical factors such as country, female ratio, average age, disease duration, probiotic quantity, and treatment duration on the primary outcome—URR, as shown in Table 2.

**Table 2:** Subgroup analyses based on female ratio, average age, probiotic quantity, and treatment duration.

Subject	Subgroup	Number of studies	*I*^2^/%	OR (95% CI)	*p* value
Female ratio	<50%	3	0	4.44 (1.94, 10.16)	0.0004
	≥50%	4	0	2.54 (1.64, 3.95)	<0.0001
Average age	<18 years old	4	0	3.04 (1.88, 4.91)	<0.00001
	≥18 years old	2	0	2.22 (1.10, 4.48)	0.03
Probiotic quantity	Single-strain probiotics	2	0	2.22 (1.10, 4.48)	0.03
	Multi-strain probiotics	5	0	3.25 (2.04, 5.19)	<0.00001
Treatment duration	2-week	2	0	5.54 (1.87, 16.37)	0.002
	4-week	4	0	2.90 (1.80, 4.67)	<0.0001

OR, odds ratio; CI, confidence interval.

For the female ratio, subgroups were divided into <50% and ≥50%. The results indicated statistically significant differences in URR for both subgroups: female ratio <50% (OR 4.44, 95% CI 1.94 to 10.16, *p* = 0.0004, *I*^2^ = 0) and ≥50% (OR 2.54, 95% CI 1.64 to 3.95, *p* < 0.0001, *I*^2^ = 0). In terms of average age, subgroups were categorized as <18 years and ≥18 years. Significant differences in URR were reported for both age groups: <18 years (OR 3.04, 95% CI 1.88 to 4.91, *p* < 0.00001, *I*^2^ = 0) and ≥18 years (OR 2.22, 95% CI 1.10 to 4.48, *p* = 0.03, *I*^2^ = 0). Regarding probiotic quantity, subgroups were defined as single-strain and multi-strain probiotics, with significant differences in URR observed in both single-strain probiotics (OR 2.22, 95% CI 1.10 to 4.48, *p* = 0.03, *I*^2^ = 0) and multi-strain probiotics (OR 3.25, 95% CI 2.04 to 5.19, *p* < 0.00001, *I*^2^ = 0) subgroups. For treatment duration, significant differences in URR were noted in the 2-week treatment duration (OR 5.54, 95% CI 1.87 to 16.37, *p* = 0.002, *I*^2^ = 0) and the 4-week treatment duration (OR 2.90, 95% CI 1.80 to 4.67, *p* < 0.0001, *I*^2^ = 0) subgroups.

These results suggest that the clinical heterogeneity arising from the female ratio, average age, probiotic quantity, and treatment duration did not have a significant impact on the results of the meta-analysis, indicating that the findings are robust. However, since there were no obvious stratifications in terms of country or disease duration among the studies included in the analysis of URR, subgroup analyses for these factors were not conducted.

### 3.6 TSA

**Figure 6:**
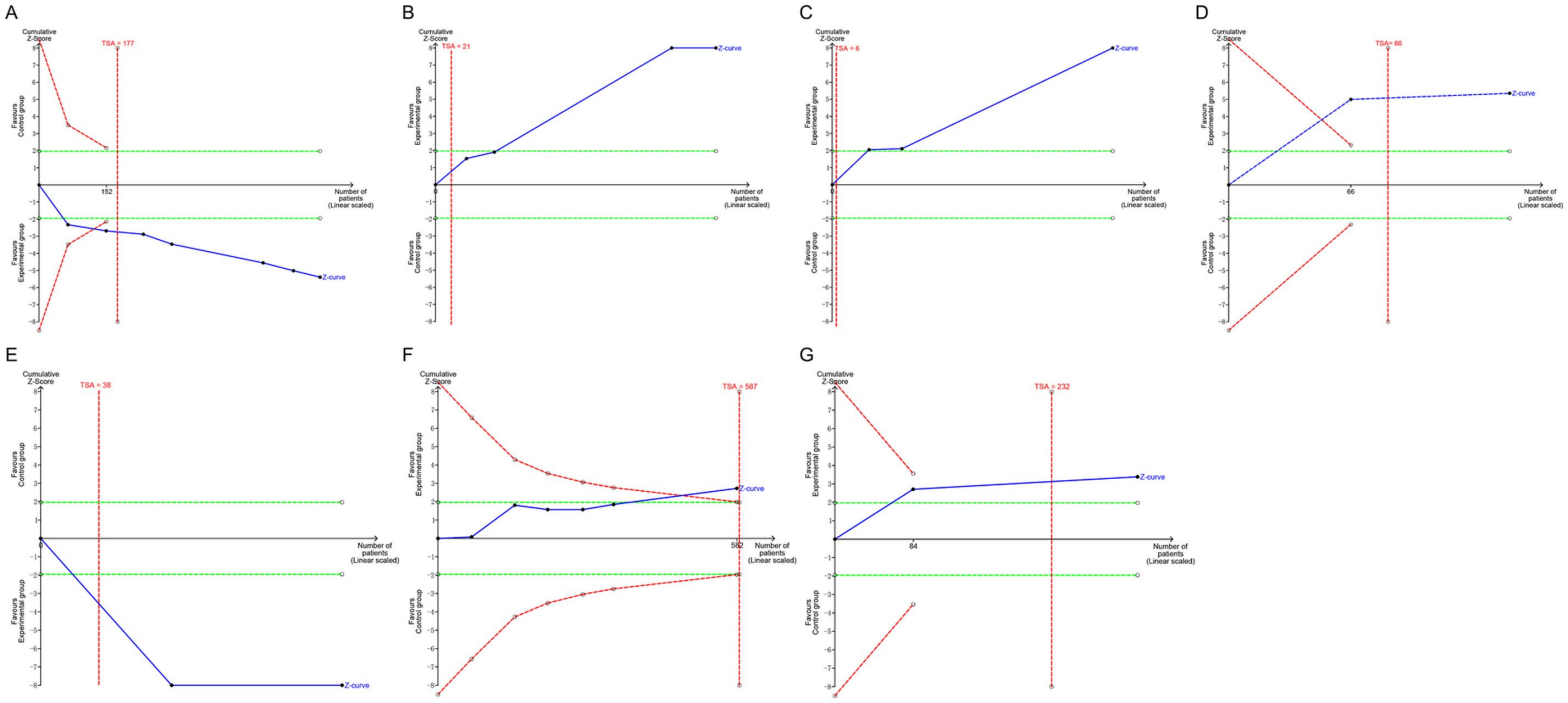
Trial sequential analyses of the efficacy and safety outcomes: **(A)** URR; **(B)** UAS7; **(C)** DLQI; **(D)** Serum IL-10 levels; **(E)** Serum IFN-γ levels; **(F)** AER; **(G)** Recurrence rate. URR, urticaria relief rate; UAS7, urticaria activity score over 7 days; DLQI, dermatology life quality index; IL-10, interleukin-10; IFN-γ, interferon-gamma; AER, adverse event rate.

The TSA demonstrated that the *Z*-value curves for UAS7, DLQI, and IFN-γ crossed the monitoring boundaries after the first study, suggesting that these outcomes reached conclusive results early on. Similarly, the *Z*-value curves for IL-10 and recurrence rate crossed the boundaries after the second study, indicating sufficient evidence for these factors. The *Z*-value curve for URR crossed the boundary after the third study, while the AER curve crossed after the sixth study, both pointing to conclusive results. These findings confirm that the meta-analysis outcomes for UAS7, DLQI, IFN-γ, IL-10, recurrence rate, URR, and AER are reliable and robust, as shown in Figure 6.

### 3.7 Publication bias

**Figure 7:**
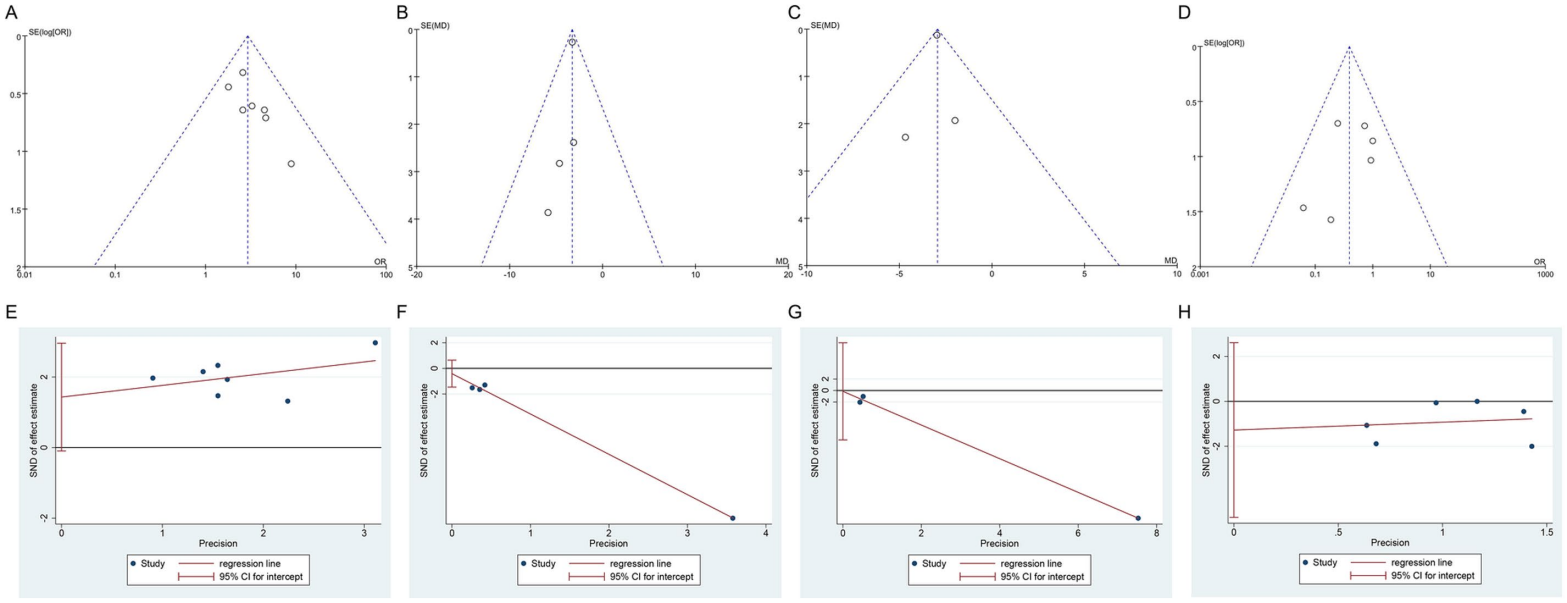
Funnel plots and Egger’s tests of publication bias: **(A)** Funnel plots of URR; **(B)** Funnel plots of UAS7; **(C)** Funnel plots of DLQI; **(D)** Funnel plots of AER; **(E)** Egger’s tests of URR; **(F)** Egger’s tests of UAS7; **(G)** Egger’s tests of DLQI; **(H)** Egger’s tests of AER. URR, urticaria relief rate; UAS7, urticaria activity score over 7 days; DLQI, dermatology life quality index; AER, adverse event rate.

Publication bias was assessed using funnel plots and Egger’s test for URR, UAS7, DLQI, and AER. Visual inspection of the funnel plots indicated potential publication bias for all four outcomes, as there was asymmetry and possible missing studies, as shown in Figures 7A–D. However, Egger’s test showed no significant evidence of publication bias: URR (*p* = 0.061), UAS7 (*p* = 0.236), DLQI (*p* = 0.886), and AER (*p* = 0.413), as shown in Figures 7E–H. Although funnel plots indicated possible bias, the statistical analysis does not confirm its presence, suggesting that publication bias likely has minimal impact on the results.

### 3.8 Certainty of evidence

The GRADE system assessments indicated that the certainty of evidence for URR, UAS7, and DLQI was characterized as low and for IL-10, IFN-γ, AER, and recurrence rate was rated as very low, reflecting some uncertainty and a need for further high-quality studies to confirm these findings, as shown in Table 3.

**Table 3:** Certainty of evidence.

Outcome	Risk of bias	Inconsistency	Indirectness	Imprecision	Others	OR/MD (95% CI)	Certainty of evidence
URR	Serious	Serious	None	None	None	2.90 (1.97, 4.28)	Low
UAS7	Serious	Serious	None	None	None	−3.29 (−3.83, −2.75)	Low
DLQI	Serious	Serious	None	None	None	−2.95 (−3.21, −2.69)	Low
IL-10	Serious	Serious	None	Serious	None	−1.47 (−2.01, −0.93)	Very low
IFN-γ	Serious	Serious	None	Serious	None	8.12 (6.95, 9.30)	Very low
AER	Serious	Serious	None	Serious	None	0.39 (0.20, 0.77)	Very low
Recurrence rate	Serious	Serious	None	Serious	None	0.29 (0.14, 0.60)	Very low

OR, odds ratio; CI, confidence interval; URR, urticaria relief rate; UAS7, urticaria activity score over 7 days; DLQI, dermatology life quality index; IL-10, interleukin-10; IFN-γ, interferon-gamma; AER, adverse event rate.

## 4 Discussion

### 4.1 Research significance and main findings

Effective control of urticaria symptoms and improvement of disease prognosis have long been challenging for clinicians (Kaplan, 2012). In recent years, the role of probiotics as an adjunct therapy has garnered increasing attention, offering new perspectives in this field (Nettis et al., 2016). To the best of our knowledge, this is the first meta-analysis and TSA that precisely evaluates the efficacy and safety of probiotics in the treatment of urticaria. Our meta-analysis indicates that, compared to antihistamines alone, the addition of probiotics significantly enhances URR and IFN-γ levels, while reducing UAS7, DLQI, IL-10, AER, and recurrence rate. Sensitivity analyses show that all results, except for AER, are robust. TSA suggests that these outcomes are conclusive, which strengthens the credibility of our findings.

### 4.2 Efficacy of probiotics in URR, UAS7, and DLQI

URR, UAS7, and DLQI are pivotal indicators for clinical evaluation of urticaria, quantifying treatment efficacy, disease activity, and burden on patients’ lives, respectively (Maurer et al., 2017; Wu et al., 2021; Wang et al., 2022). Our findings support that the supplementation of probiotics leads to benefits in outcomes such as URR, UAS7, and DLQI. Previous meta-analyses and RCTs provide support for the results of this study. First, a meta-analysis conducted by Fu et al. (2023) found that the addition of probiotics to antihistamines significantly improved the treatment effect of urticaria (RR 1.09, 95% CI 1.03–1.16). Their results suggested that probiotics could promote the relief of urticaria symptoms, although evidence of improvement in UAS7 and DLQI was not specifically reported. Second, a RCT by Bi et al. (2021) demonstrated that, compared with desloratadine alone, the combination of multi-strain probiotics (comprising *Lactobacillus gasseri*, *Lactobacillus salivarius*, *Lactobacillus johnsonii*, *Lactobacillus paracasei*, *Lactobacillus reuteri*, and *Bifidobacterium animalis*) and desloratadine significantly reduced wheal size and attack frequency in children with urticaria, thereby achieving a higher URR. Desloratadine, a second-generation H_1_-antihistamine, is widely used in the treatment of allergic diseases such as urticaria due to its high H_1_ receptor selectivity and favorable safety profile. Although a recent *in vitro* study reported that desloratadine exhibits antimicrobial and antibiofilm activity against multidrug-resistant *Acinetobacter baumannii* at concentrations exceeding 64 μg/mL, these effects were observed at levels far above the plasma concentrations achieved with standard therapeutic dosing (Eduvirgem et al., 2023). Therefore, current evidence does not support any significant interaction or interference between desloratadine and probiotic strains at clinically relevant concentrations. Additionally, RCTs by Ye et al. (2018) and Fu et al. (2020) reported that the combination of probiotic yeast with cetirizine, a well-established second-generation antihistamine, or with rupatadine, a newer-generation antihistamine, also significantly improved the URR. RCTs by Dabaghzadeh et al. (2023) and Wang et al. (2023) further indicated that the addition of multi-strain probiotics to standard treatment regimens led to greater reductions in UAS7 and DLQI scores compared to conventional therapy alone. The probiotic formulation used by Dabaghzadeh et al. (2023) included *Lactobacillus casei*, *Lactobacillus acidophilus*, *Lactobacillus bulgaricus*, *Lactobacillus rhamnosus*, *Bifidobacterium breve*, *Bifidobacterium longum*, and *Streptococcus thermophilus*. The formulation used by Wang et al. (2023) contained *Enterococcus faecium* and *Bacillus subtilis*. These pieces of evidence point to the benefits of probiotics in improving URR, UAS7, and DLQI in patients with urticaria, which supports our findings. Interestingly, Atefi et al. (2022) reported that multi-strain probiotics (comprising *Lactobacillus rhamnosus*, *Lactobacillus casei*, *Lactobacillus acidophilus*, *Bifidobacterium breve*, *Lactobacillus bulgaricus*, *Bifidobacterium longum*, *Streptococcus thermophilus*) reduced DLQI in patients with urticaria, but had no significant effect on UAS7 (*p* = 0.137). This discrepancy in UAS7 outcomes may be related to the background treatment with antihistamines. In the study by Atefi et al. (2022), patients received a combination of two antihistamines, whereas other included studies used only one. The concurrent use of two antihistamines likely enhanced the reduction in UAS7 to a greater extent, potentially diminishing the additional measurable effect of probiotics on disease activity. In summary, probiotics effectively reduce clinical symptoms, disease activity and the burden of life for patients with urticaria. Supplementary Table S2 summarizes the probiotic formulations used in the included studies, detailing their strain composition and availability.

Notably, our meta-analysis clarified for the first time the effects of probiotics on IL-10 and IFN-γ in patients with urticaria, filling the gap in previous meta-analyses. In previous clinical trials, Li et al. (2013) and Chen et al. (2013) reported that the combination of *Bifidobacterium quadruple* viable tablets and antihistamines could significantly reduce the serum IL-10 level and increase the serum IFN-γ level in patients with urticaria, supporting our findings. Additionally, the clinical trial of Wang et al. (2023) reported that probiotics reduced the serum IL-6, TNF-α and IgE as well as the peripheral blood CD8+ level in patients with chronic spontaneous urticaria. Animal experiments by Hoa et al. (2024) demonstrated that *Bifidobacterium longum* KACC91563 alleviated allergic contact dermatitis in mice by downregulating serum levels of key pro-inflammatory and Th2/Th17 associated cytokines, including IgE, IL-4, IL-5, IL-13, IL-17, and TNF-α. These findings suggest that probiotics may modulate allergic immune responses through anti-inflammatory and immunoregulatory pathways. Further studies indicate that the regulatory effects of probiotics on immunity and inflammation are achieved by remodeling the gut microbiota and restoring the intestinal barrier (Zheng et al., 2023). For one thing, patients with urticaria are often accompanied by gut microbiota imbalance, which is characterized by a decrease in the abundance of *Lactobacillus*, *Bifidobacterium*, and *Lachnospira* (Ćesić et al., 2023; Rezazadeh et al., 2018). The supplementation with specific probiotic strains, such as *Lactobacillus rhamnosus*, *Lactobacillus plantarum*, *Lactobacillus salivarius*, *Bifidobacterium longum*, *Bifidobacterium breve*, *Bifidobacterium bifidum*, and *Enterococcus faecium*, has been shown to restore gut microbial balance by increasing the abundance of beneficial taxa and reducing proteobacterial overgrowth (Smolinska et al., 2025). For another, probiotics such as *Lactobacillus rhamnosus*, *Lactobacillus plantarum*, *Limosilactobacillus reuteri*, *Lactobacillus acidophilus*, *Streptococcus thermophilus*, *Bifidobacterium longum*, and *Bifidobacterium bifidum* have been shown to enhance the integrity of the intestinal mucosal barrier, thereby reducing the translocation of potential pathogens such as Escherichia and their metabolites, and ultimately alleviating inflammation (Rao and Samak, 2013). In summary, probiotics may alleviate immune dysregulation and inflammatory response by rebuilding the intestinal microbiota and enhancing the intestinal barrier function, ultimately improving the prognosis of urticaria.

### 4.3 Efficacy of probiotics in AER and recurrence rate

Our meta-analysis indicated that probiotics combined with antihistamines significantly reduced AER by 61% and recurrence rate by 71% compared with antihistamines alone. Regarding recurrence rates, Wang et al. (2023) and Ye et al. (2018) respectively reported that multi-strain and single-strain probiotics reduced urticaria recurrence rate, which supports our findings. Sensitivity analyses and TSA suggested that the results of recurrence rate were robust and conclusive, further emphasizing the value of probiotics in reducing recurrence rate. Regarding adverse events, although TSA indicates that the meta-analysis results for the AER were conclusive, sensitivity analyses showed that the robustness of this conclusion was questionable. Specifically, after excluding the study of Wang et al. (2023), the AER of the two groups was no longer statistically significant (OR 0.50, 95% CI 0.24–1.05, *p* = 0.07). We speculate that the high sensitivity of AER in the overall analysis may be due to the small sample size, especially as 41.2% of the participants were from the study of Wang et al. (2023). After excluding this study, the meta-analysis included only 342 participants, which may be insufficient to detect a true difference in AER. Notably, none of the included studies reported statistical differences in AER (Li et al., 2013; Can et al., 2024; Dabaghzadeh et al., 2023; Bi et al., 2021; Atefi et al., 2022), but the meta-analysis identified statistical differences. This further supports our speculation that the negative findings may be attributed to the small sample size. Although the evidence that probiotics reduce AER needs further supplementation and strengthening, all available findings suggest that probiotics do not increase additional adverse events. In summary, probiotics are effective in reducing the recurrence rate of urticaria and may potentially decrease adverse events during treatment.

### 4.4 Implications for practice and research

This meta-analysis highlights the potential of probiotics as a safe and affordable adjunct therapy to antihistamines in the management of urticaria. Moreover, our findings reinforce the plausibility of the gut–skin axis as a biological basis for therapeutic interventions (Sinha et al., 2021). The observed benefits of probiotic supplementation in urticaria support the hypothesis that modulating gut microbiota may contribute to the improvement of skin inflammatory diseases. This provides a promising foundation for exploring the gut–skin axis as a potential therapeutic target in future research. Given the low cost, good safety profile, and ease of administration of probiotics, their use may represent a scalable and practical strategy to enhance patient quality of life and reduce treatment burden, especially in resource-limited settings. These findings provide preliminary evidence supporting the integration of probiotic-based adjunctive therapy into the broader clinical management framework for urticaria.

### 4.5 Limitations and prospects

While this meta-analysis provides preliminary evidence supporting probiotics as a complementary therapy for urticaria, several important limitations must be acknowledged. First, the majority of included studies were conducted in China, which may introduce regional and publication bias, limiting the applicability of the findings to broader, more diverse populations. Second, potential bias may have arisen during data synthesis due to the relatively small number of available studies for each outcome. This limitation reduces the statistical power of the meta-analyses and raises concerns about the robustness and reliability of the conclusions, particularly for outcomes supported by only two or three trials. Third, there was potential heterogeneity across studies in terms of probiotic strains, dosages, treatment durations, and study designs. Although subgroup analyses suggested that both single-strain and multi-strain probiotics confer benefits, the optimal strain combinations and treatment regimens remain undefined and cannot be generalized based on the current evidence. Fourth, the certainty of evidence for some immune-related outcomes, such as IL-10, IFN-γ, and recurrence rate, was graded as very low, underscoring the need for higher-quality trials. Additionally, the sensitivity analysis revealed instability in the findings related to adverse event rates, suggesting that the current safety conclusions should be interpreted with caution.

Future research should address these limitations by conducting large-scale, rigorously designed randomized controlled trials in more diverse geographical and demographic settings. Standardizing probiotic formulations, dosages, and treatment durations will be essential for developing reliable therapeutic protocols. Moreover, future studies should aim to identify the most effective probiotic strains or combinations thereof, and generate higher-certainty evidence for both clinical and immunological outcomes. Ongoing safety monitoring and robust analytical methods will also be critical to ensure the reliable integration of probiotics into the clinical management of urticaria.

## 5 Conclusion

Probiotics not only improve clinical symptoms in patients with urticaria but may also reduce adverse event and recurrence rates. This highlights the potential of probiotics as a complementary treatment for urticaria. However, due to the risk of bias in existing studies, these findings require validation through high-quality clinical trials.

## Data Availability

The original contributions presented in the study are included in the article/Supplementary material, further inquiries can be directed to the corresponding authors.

## Footnotes

1
http://www.cnki.net

2
http://www.wanfangdata.com.cn

3
http://www.cqvip.com

4
http://training.cochrane.org/online-learning/core-software-cochrane-reviews/revman
