# Supplementary figures and images for: Efficacy and safety of probiotics as a complementary treatment for urticaria: a systematic review and meta-analysis
Source: Front Microbiol. 2025 Dec 10;16:1634990. doi: 10.3389/fmicb.2025.1634990 (PMC12728354; doi:10.3389/fmicb.2025.1634990)

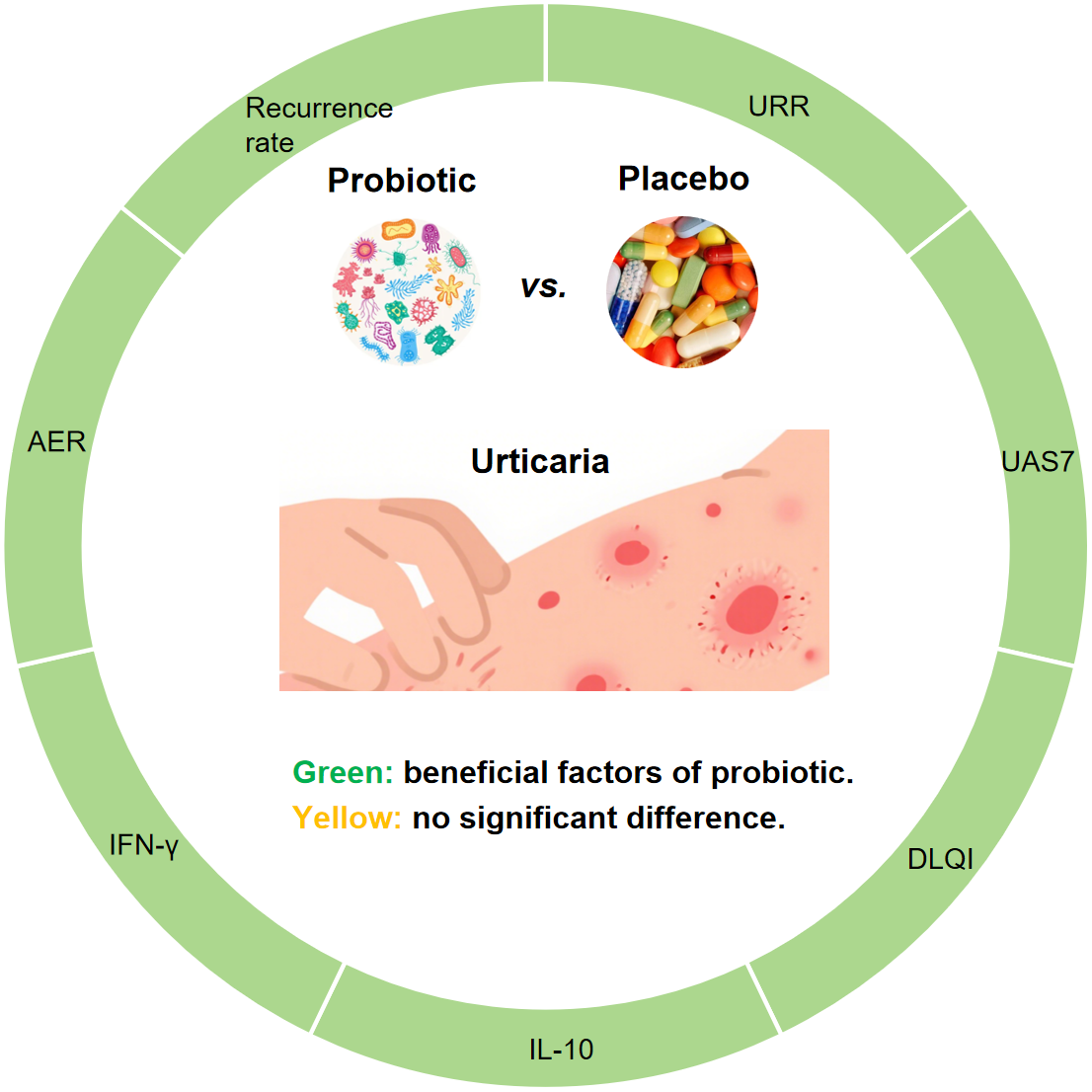

Supplement: Supplementary file 1 [file Data_Sheet_1.ZIP › Figures/Figure 1.jpg]

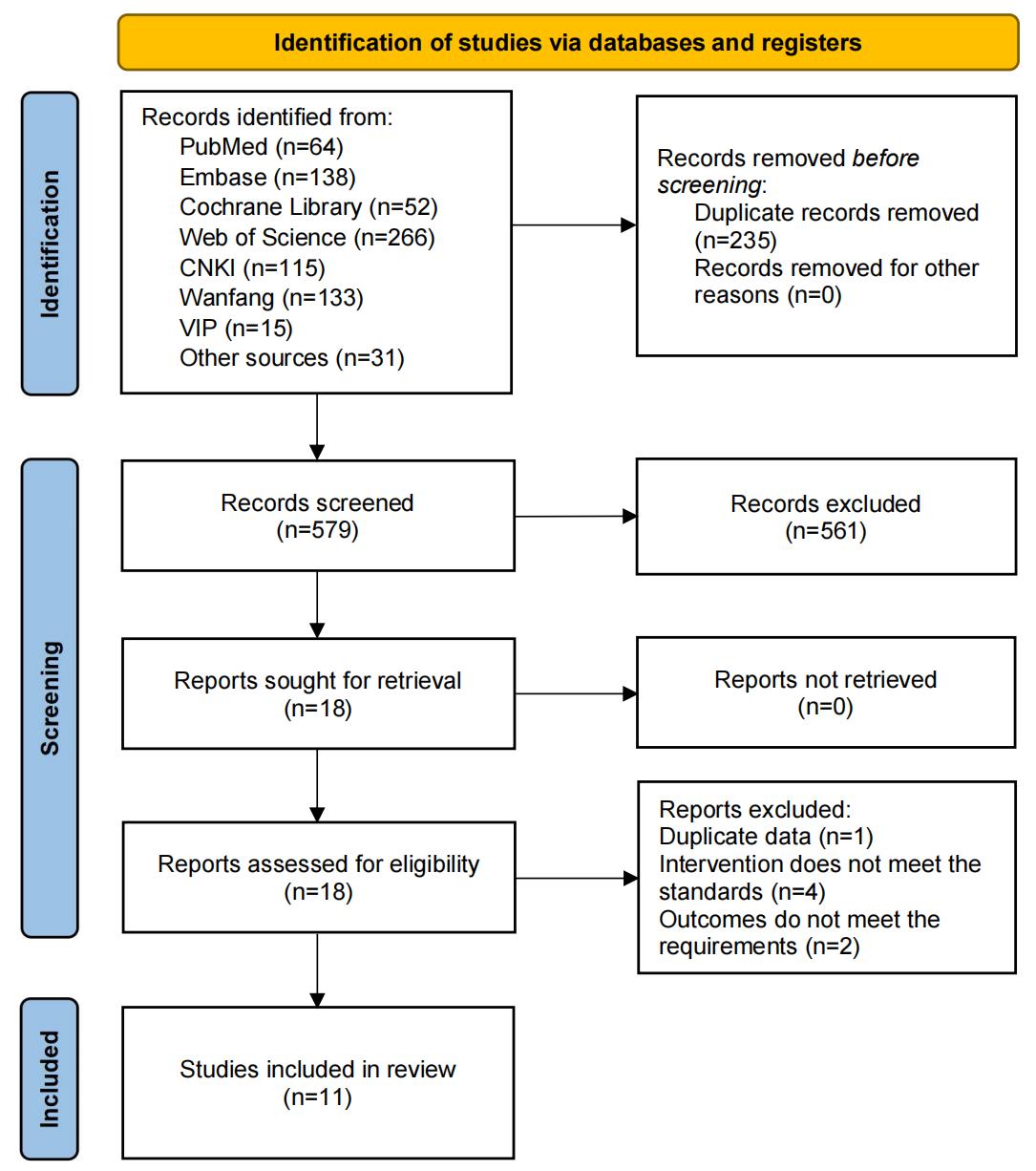

Supplement: Supplementary file 1 [file Data_Sheet_1.ZIP › Figures/Figure 2.jpg]

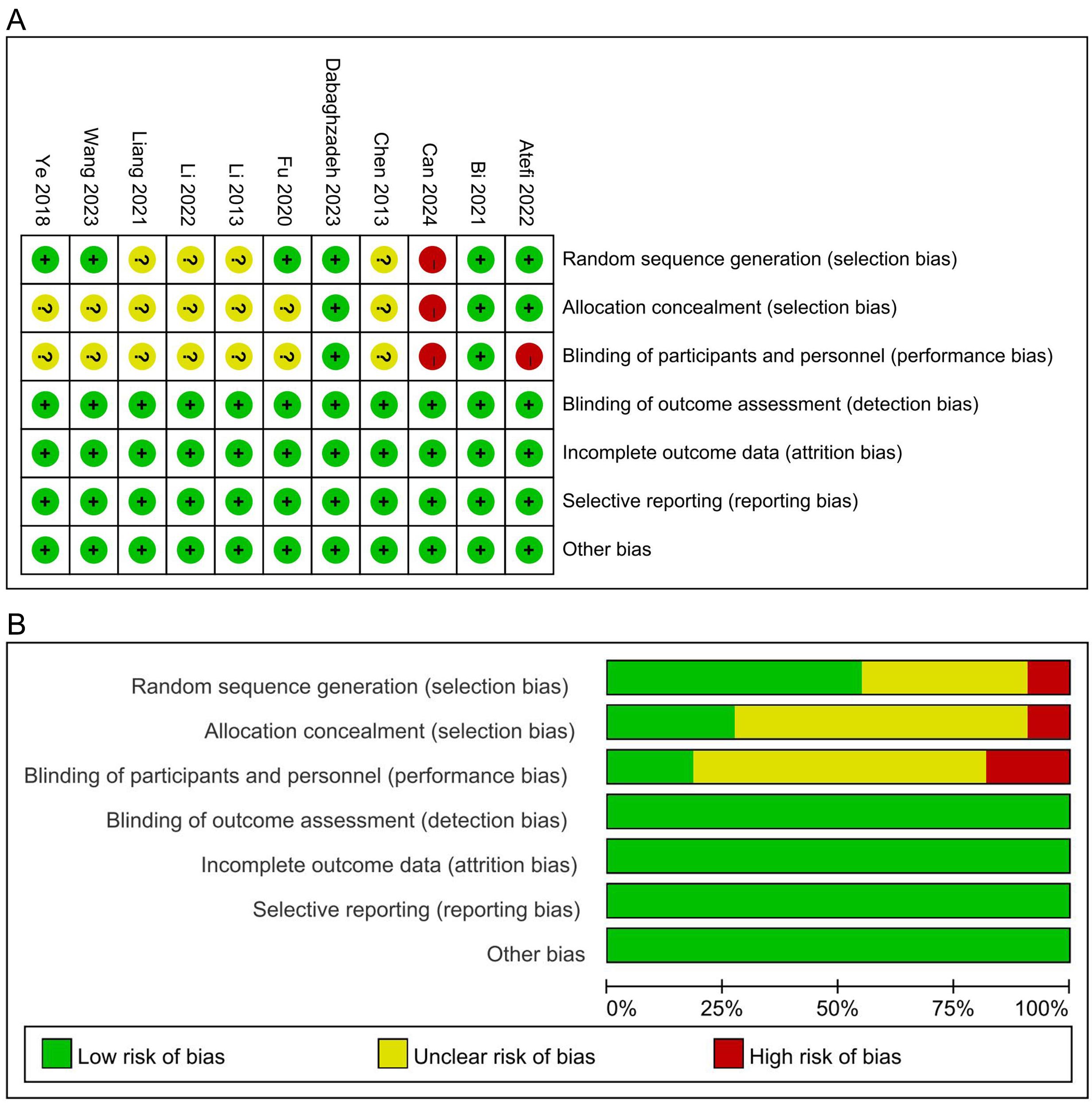

Supplement: Supplementary file 1 [file Data_Sheet_1.ZIP › Figures/Figure 3.jpg]

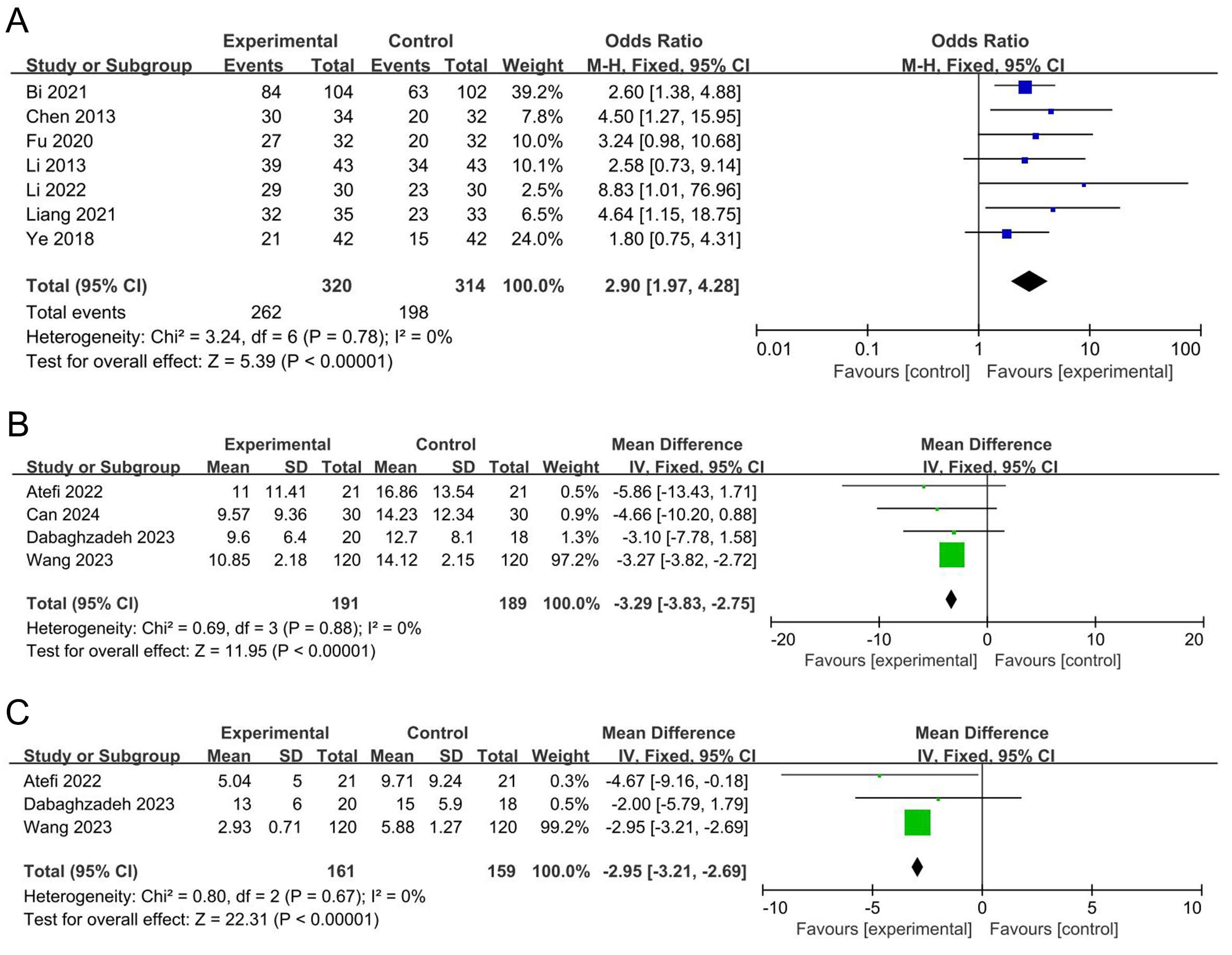

Supplement: Supplementary file 1 [file Data_Sheet_1.ZIP › Figures/Figure 4.jpg]

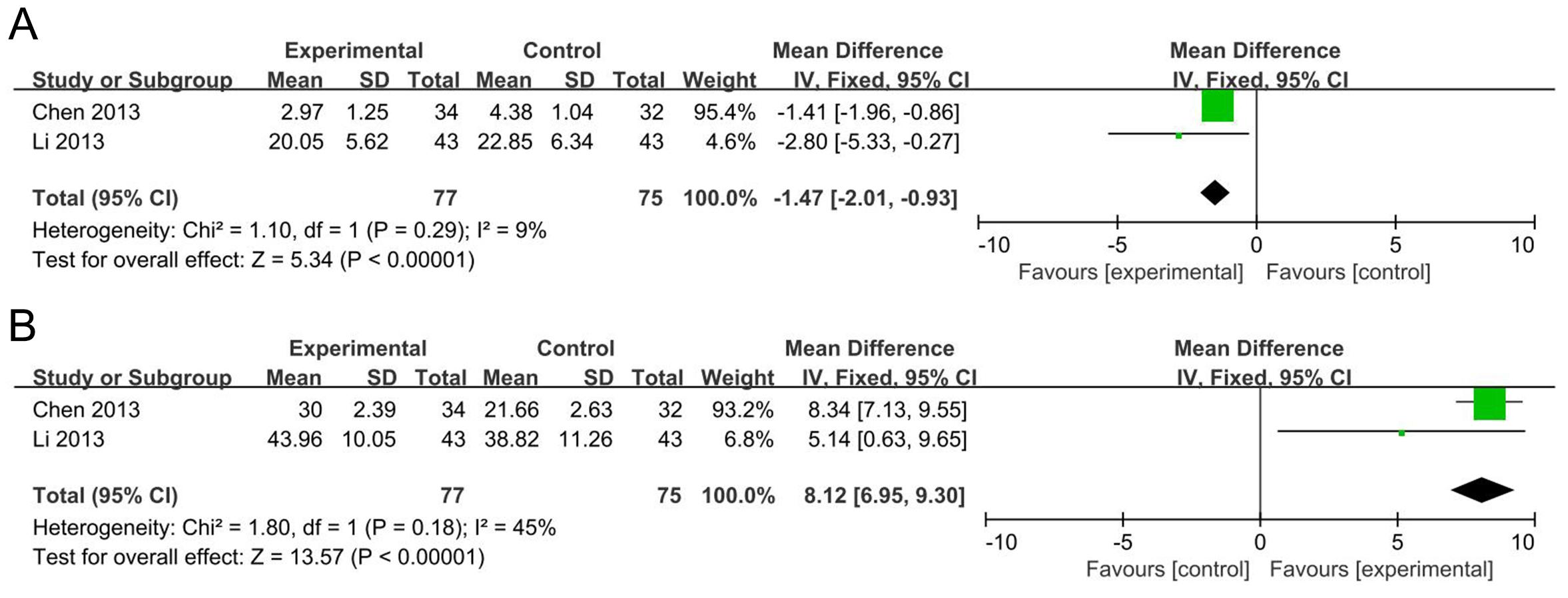

Supplement: Supplementary file 1 [file Data_Sheet_1.ZIP › Figures/Figure 5.jpg]

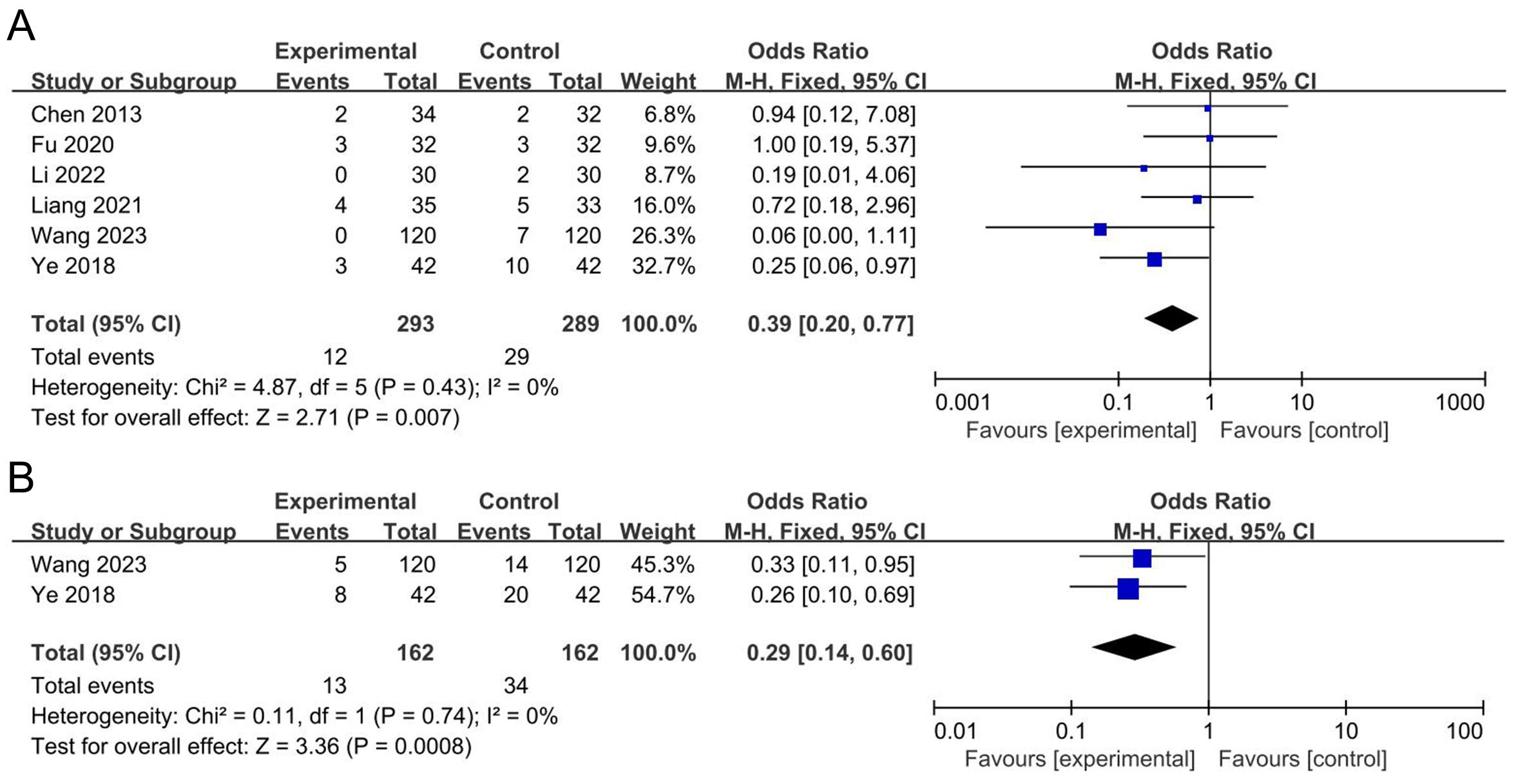

Supplement: Supplementary file 1 [file Data_Sheet_1.ZIP › Figures/Figure 6.jpg]

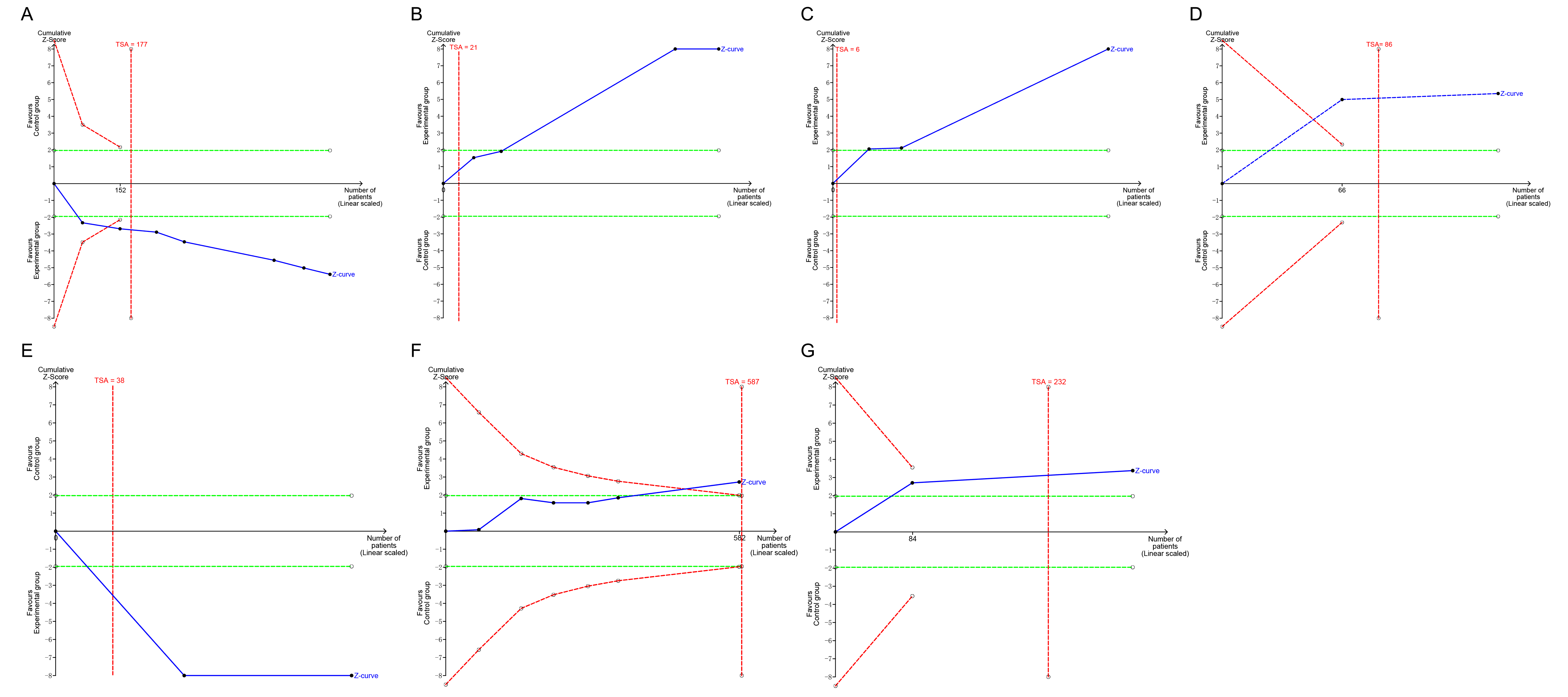

Supplement: Supplementary file 1 [file Data_Sheet_1.ZIP › Figures/Figure 7.jpg]

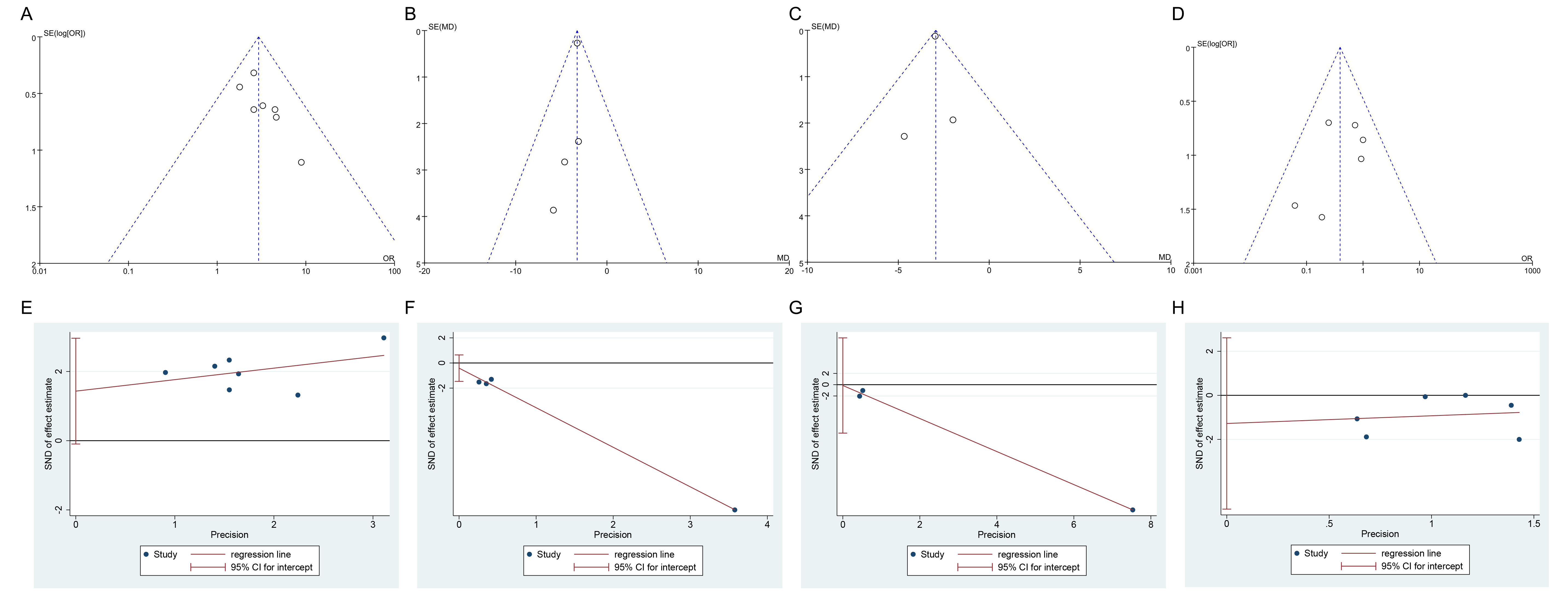

Supplement: Supplementary file 1 [file Data_Sheet_1.ZIP › Figures/Figure 8.jpg]
